# VT – ohne strukturelle Herzerkrankung: Historischer Überblick

**DOI:** 10.1007/s00399-024-01007-z

**Published:** 2024-02-26

**Authors:** Christian-Hendrik Heeger, Roland Richard Tilz

**Affiliations:** 1 Department für Rhythmologie, Abteilung für Kardiologie & Internistische Intensivmedizin, Asklepios Klinik Altona, Paul-Ehrlich-Straße 1, 22763 Hamburg, Deutschland; 2 Klinik für Rhythmologie, Universitäres Herzzentrum Lübeck, Universitätsklinikum Schleswig-Holstein (UKSH), Ratzeburger Allee 160, 23538 Lübeck, Deutschland

**Keywords:** Ventrikuläre Tachykardie, Idiopathische ventrikuläre Arrhythmie, Ventrikuläre Extrasystolie, Katheterablation, Ventricular tachycardia, Idiopathic ventricular arrhythmia, Premature ventricular contractions, Catheter ablation

## Abstract

Dieser Beitrag widmet sich dem Thema ventrikuläre Arrhythmie ohne vorliegende strukturelle Herzerkrankung. Die zugrunde liegenden Ursachen sind vielfältig und noch nicht komplett verstanden. Beginnend bei der Erstbeschreibung dieser Art der Arrhythmie nähern wir uns über die Diagnostik der Therapie mittels Medikamenten und schließlich der Katheterablation von den Anfängen bis hin zur den modernsten aktuellen und zukünftigen Methoden.

## Abstract (en)

This article focuses on ventricular arrythmias without evidence for structural heart disease. There are many different reasons for this type of arrythmia and there is still a gap of knowledge. Starting with the first description of this disease, we present the diagnosis and management with medication, and finally catheter ablation procedures from the beginning to how it is currently treated and how it possibly will be treated in the near future.

Die Geschichte der ventrikulären Tachykardien (VT) ohne strukturelle Herzerkrankung ist relativ jung, da diese Art von Herzrhythmusstörung erst in den letzten Jahrzehnten intensiver erforscht wurde und bis heute auch noch längst nicht komplett verstanden ist. Eine VT ist eine Form der Herzrhythmusstörung, bei der der Herzrhythmus meist regelmäßig und stark beschleunigt ist. In den meisten Fällen tritt diese Art von Tachykardie bei Menschen mit strukturellen Herzerkrankungen auf, wie zum Beispiel nach einem Herzinfarkt, nach Herzmuskelentzündung (Myokarditis) oder auch bei angeborenen Herzfehlern [1]. Diese strukturellen Formen der ventrikulären Arrhythmien werden in diesem Werk im Kapitel „VT bei struktureller Herzerkrankung“ beschrieben und der Vollständigkeit halber hier nur erwähnt. In den 1960er und 1970er-Jahren begannen Forscher festzustellen, dass es neben den klassischen Patienten nach einem Herzinfarkt und vorliegender struktureller Herzerkrankung, auch Patienten gab, die unter VT und ventrikulärer Extrasystolie (VES) litten, obwohl keine strukturelle Herzerkrankungen nachweisbar war. Die Patienten hatten in Ruhe ein normales 12-Kanal-Elektrokardiogramm (EKG) und eine normale Herzfunktion in der Echokardiographie. Sie wiesen jedoch regelmäßig auftretende schnelle Herzrhythmusstörungen auf (VT und repetitive VES), die zum Teil nur Palpitationen verursachten, es waren aber auch Synkopen und zum Teil lebensbedrohliche Zustände nachweisbar. In den folgenden Jahrzehnten wurden verschiedene Ursachen für die VT ohne strukturelle Herzerkrankung identifiziert. Eine wichtige Entdeckung war, dass bestimmte elektrophysiologische Eigenschaften des Herzens eine Rolle spielen können. Zum Beispiel können Veränderungen in den Ionenkanälen des Herzens zu einer gestörten Signalübertragung führen, was zu einer Tachykardie führt. Ein weiterer wichtiger Faktor für die Entwicklung von VT ohne strukturelle Herzerkrankung ist Stress. Psychischer oder körperlicher Stress kann zu einer erhöhten Ausschüttung von Stresshormonen wie Adrenalin führen. Diese Hormone können das elektrische System des Herzens beeinflussen und Herzrhythmusstörungen auslösen. Die Behandlung der VT ohne strukturelle Herzerkrankung hat sich im Laufe der Zeit deutlich weiterentwickelt. Die Verwendung von Antiarrhythmika, die Katheterablation und in seltenen Fällen auch die Verwendung von implantierbaren Defibrillatoren (ICD) sind heute gängige Methoden, um die Arrhythmie zu kontrollieren und das Risiko für lebensbedrohliche Komplikationen zu verringern. Insgesamt hat die Erforschung der VT ohne strukturelle Herzerkrankung dazu beigetragen, das Verständnis der Herzrhythmusstörungen zu erweitern und die Möglichkeit von präventiven Behandlungsmaßnahmen zu erforschen. Durch fortlaufende Forschung und verbesserte Technologien hofft man, die Behandlungsmöglichkeiten und die Lebensqualität der betroffenen Patienten weiter zu verbessern. Es ist hier wichtig zu erwähnen das sich historisch gesehen die Zuordnung von einigen Syndromen und ursächlichen Erkrankungen die zu einer VT führen können im Laufe der Zeit mit der Entwicklung von Bildgebung, Histologie und Molekularbiologie gewandelt hat. Zum Beispiel wurden das Brugada-Syndrom und die Arrhythmogene rechtsventrikuläre Dysplasie (ARVD) lange als idiopathisch, ohne Vorliegen einer strukturellen Herzerkrankung, bezeichnet. Heute wissen wir allerdings, dass hier auf zellulärer Ebene auch struktureller Komponenten beteiligt sind [2]. Die folgenden Abschnitte sollen einen Überblick über die Ursachen und Behandlungsmöglichkeiten von VT ohne strukturelle Herzerkrankung geben. Insgesamt ist die Prognose der VT ohne strukturelle Herzerkrankung deutlich besser als im Fall einer vorliegenden strukturellen Herzerkrankung [3]. Jedoch weisen einige Beobachtungsstudien darauf hin, dass ventrikuläre Arrhythmien auch bei fehlender strukturellerer Herzerkrankung mit einer erhöhten Mortalität einher gehen können [4]. Daher sollte auch bei Fehlen einer strukturellen Herzerkrankung bei ventrikulärer Arrhythmien immer eine erhöhte Wachsamkeit erfolgen und weitere Diagnostik und ggf. Therapie nach sich ziehen.

## Ursachen – VT ohne strukturelle Herzerkrankung

Die genauen Ursachen für VT ohne strukturelle Herzerkrankung sind noch nicht vollständig verstanden. Es gibt verschiedene mögliche Ursachen und Theorien für die Entstehung von VT bei Fehlen einer strukturellen Herzerkrankung:

1. Vererbung: Einige genetische Mutationen können zu elektrischen Störungen im Herzen führen, die zu ventrikulärer Arrhythmie führen können (Brugada-Syndrom, Long-QT-Syndrom, ARVD, katecholaminerge polymorphe ventrikuläre Tachykardie [CPVT]; [5, 6]).
2. Elektrolytungleichgewichte: Ungleichgewichte von Elektrolyten wie Kalzium, Kalium oder Magnesium im Blut können zu Funktionsstörungen des Herzens führen und VT verursachen.
3. Drogen oder Medikamente: Bestimmte Medikamente oder Drogen können zu ventrikulärer Arrhythmie verursachen können. Dazu gehören zum Beispiel bestimmte Antiarrhythmika, Antiemetika oder Stimulanzien.
4. Sympathikusüberaktivität: Ein überaktives sympathisches Nervensystem kann zu einer erhöhten Herzfrequenz führen und die Wahrscheinlichkeit von VT erhöhen.
5. Hormonelle Veränderungen: Hormonelle Veränderungen, wie sie während der Schwangerschaft auftreten können, können zu VT ohne strukturelle Herzerkrankung führen.

Zur zugrundeliegenden Pathophysiologie gibt es mehrere Theorien:

Zunächst kommt es durch eine abnorme Erregungsbildung durch eine getriggerte Aktivität zu einer frühen Spätdepolarisation oder einer verzögerten Spätdepolarisation. Beispiel für eine frühe Spätdepolarisation sind die Long-QT-Syndrome [7]. Die sog. fokale Automatie kann ebenfalls zur Entstehung von VT und VES führen. Typisch für diese Art der Pathophysiologie sind die ventrikulären Arrhythmien aus dem rechtsventrikulären Ausflusstrakt (RVOT) und linksventrikulären Ausflusstrakt (LVOT).

Es ist wichtig zu beachten, dass die VT ohne strukturelle Herzerkrankung oft als idiopathisch bezeichnet wird, was bedeutet, dass die genaue Ursache unbekannt ist. In einigen Fällen kann eine sorgfältige Untersuchung durch Expert:innen helfen, die zugrundeliegende Ursache zu identifizieren, während in anderen Fällen die genaue Ursache möglicherweise nicht festgestellt werden kann. Die Behandlung richtet sich in der Regel nach den Symptomen, einer eingeschränkten linksventrikulären Funktion (LV) sowie nach der Häufigkeit der Episoden [8].

## Therapie – VT ohne strukturelle Herzerkrankung

Die Indikation zur Therapie von ventrikulären Arrhythmien ohne strukturelle Herzerkrankung beruht auf der Symptomatik und/oder einer vorliegenden eingeschränkten LV-Funktion aber auch auf der EKG-Morphologie und damit der Lokalisation des Ursprungs und Häufigkeit der Arrhythmie [8]. Historisch gesehen stand zur Therapie dieser Arrhythmien lediglich eine medikamentöse Therapie mittels β‑Blockern oder Kalziumantagonisten vom Non-Dihydropyridin-Typ zur Verfügung [9]. In einer 2002 publizierten Studie konnte hier allerdings ein lediglich leicht über dem Placeboeffekt liegender Effekt beobachtet werden [9].

Andere Antiarrhythmika (Sotalol, Flecainid, Propafenon oder Amiodaron) haben eine höhere Wirksamkeit, besitzen aber auch ein deutlich höheres Risiko für proarrhythmische Effekte [10].

Mit der Entdeckung der Katheterablation durch Melvin Scheinman (San Francisco, USA) im Jahr 1981 stand eine neuartige Therapieoption zur Behandlung von Arrhythmien zur Verfügung [11]. Zunächst waren die Ziele der Hochfrequenzstromablation Teile des Reizleitungssystems zur Behandlung der Symptome von Vorhofflimmern [11]. Schnell wurde jedoch auch der hohe Nutzen für die Behandlung weiterer Arrhythmien, wie z. B. die Katheterablation von akzessorischen Leitungsbahnen beim Wolff-Parkinson-White-Syndrom, durch die Arbeitsgruppe um Melvin Scheinman und Fred Morady erkannt und 1984 erfolgreich eingesetzt [12]. Im Jahre 1988 gelang es dann Kuck et al. (Hamburg, Deutschland) erstmalig, die Katheterablation zur Therapie akzessorischer Leitungsbahnen im linken Herz-Kreislauf-System erfolgreich zu behandeln [13–15].

Seit diesen ersten erfolgreichen Fallberichten hat sich die Katheterablation auch auf die Behandlung weiterer Arrhythmien ausgedehnt. Die ersten Publikationen die sich mit der Katheterablation von ventrikulären Tachykardien bei Patienten ohne strukturelle Herzerkrankung beschäftigten, lassen sich auf die Jahre 1992–1995 zurückdatieren [16, 17].

**Figure 1:**
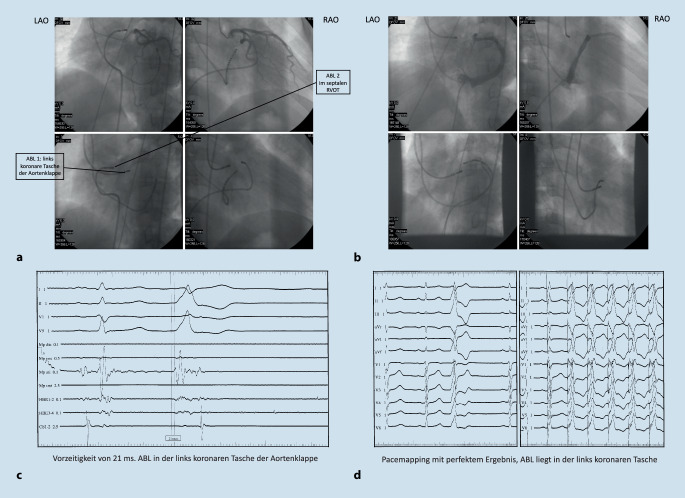

**Figure 2:**
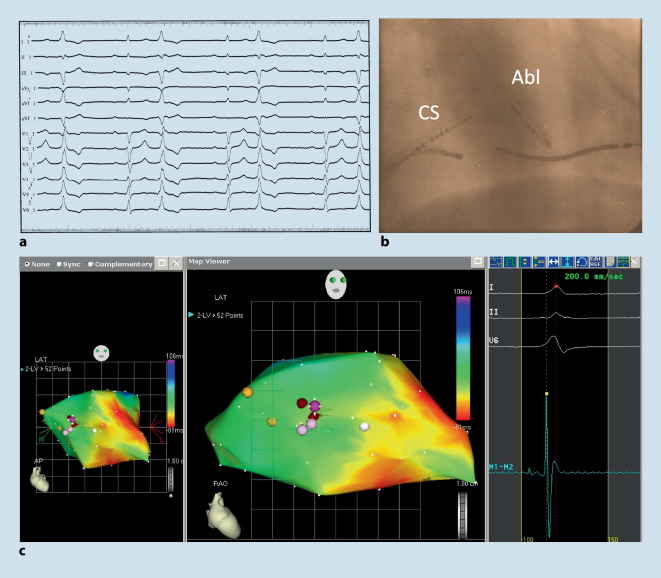

**Figure 3:**
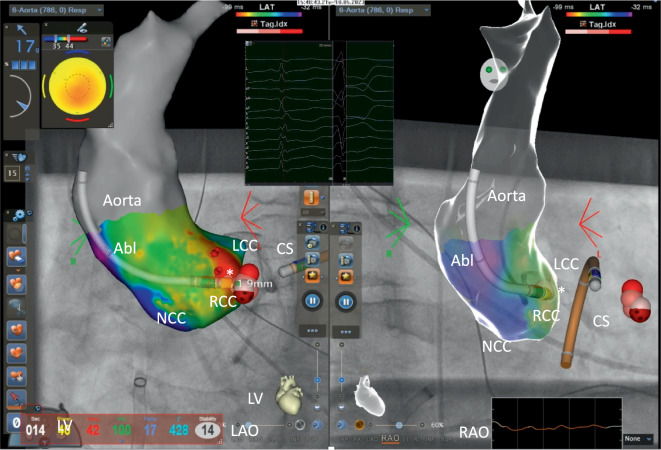

So berichteten 1992 Klein et al. aus Indiana (Indianapolis, USA) von 16 Patienten ohne Hinweise auf eine strukturelle Herzerkrankung aber mit rezidivierenden VT, die sie mittels Hochfrequenzstromablation erfolgreich behandelten. Bereits in dieser Arbeit wurden Pacemapping und Aktivierungsmapping zur Lokalisation des Ursprungs der Arrhythmie erfolgreich eingesetzt. Die Lokalisation der Arrhythmien konnte hier wie folgt identifiziert werden (*n* = 12: RVOT; *n* = 3 Trikuspidalklappen Annulus; *n* = 1 LV-Septum). Insgesamt konnten 15/16 Patienten erfolgreich behandelt werden, es wurden keine schwerwiegenden Komplikationen berichtet [17]. Zhu et al. aus Houston (Texas, USA) berichteten 1995 erstmalig über eine Serie von 10 Patienten mit idiopathischen medikamentenresistenten VES, die mittels Katheterablation behandelt wurden. Die Lokalisation hier war ebenfalls zu 90 % im RVOT und 10 % im LVOT zu finden [16]. Trotz dieser anfänglichen Erfolge wurden diese ersten Berichte zur Therapie von idiopathischen ventrikulären Arrhythmien mittels Katheterablation im Feld der Rhythmologie zunächst eher kritisch gesehen und als „*A Therapy in Search of a Disease“ bezeichnet* [18]. Es ist wichtig zu erwähnen, dass die diese Anfänge der Katheterablation komplett ohne steuerbare und gekühlte Katheter, ohne steuerbare Schleusen und auch ohne 3D-Mapping-Systeme erfolgten (Abb. 1). Mit der Einführung dieser zusätzlichen Verbesserungen war es möglich, auch immer komplexere und auch schwer zu erreichenden Ursprungsorte der ventrikulären Arrhythmien zu identifizieren (Abb. 2). So wurde neben dem RVOT als häufiger Ursprungsort für idiopathische ventrikulären Arrhythmien auch der LVOT einschließlich dem Bereich des Aortensinus (Abb. 3) und des epikardialen Umfeldes als zwar seltene, aber dennoch wichtige Regionen identifiziert, die ebenfalls mittels Katheterablation erfolgreich behandelt werden können [19]. Die ersten Berichte einer erfolgreichen Ablation von ventrikulären Arrhythmien aus dem Aortensinus stammen von 1999 aus Fukuoka, Japan (Shimoike, E. et al.) [20]. Eine genaue Charakterisierung und Differenzierung innerhalb des Aortensinus erfolgte durch die Arbeitsgruppe von K.-H. Kuck und F. Ouyang aus Hamburg, Deutschland [21–23]. Neben dem Aortensinus kann auch der pulmonalarterielle Sinus Ursprung für ventrikuläre Arrhythmien sein [24]. Die Charakterisierung und Beschreiung der erfolgreichen Ablation aus diesem Bereich erfolgte durch eine gemeinsame Arbeit einer Arbeitsgruppe aus Guangdong, China, und Hamburg, Deutschland [25].

Die Katheterablation ist bei symptomatischen, monomorphen VES/VT eine sichere und effektive Alternative zur antiarrhythmischen Medikation. In den aktuellen Leitlinien zur Therapie ventrikulärer Arrhythmien ohne Hinweise auf eine strukturelle Herzerkrankung wird die Katheterablation je nach Vorliegen weiterer Faktoren mit einer Klasse I bis IIb empfohlen [8].

## Ausblick in die Zukunft

**Figure 4:**
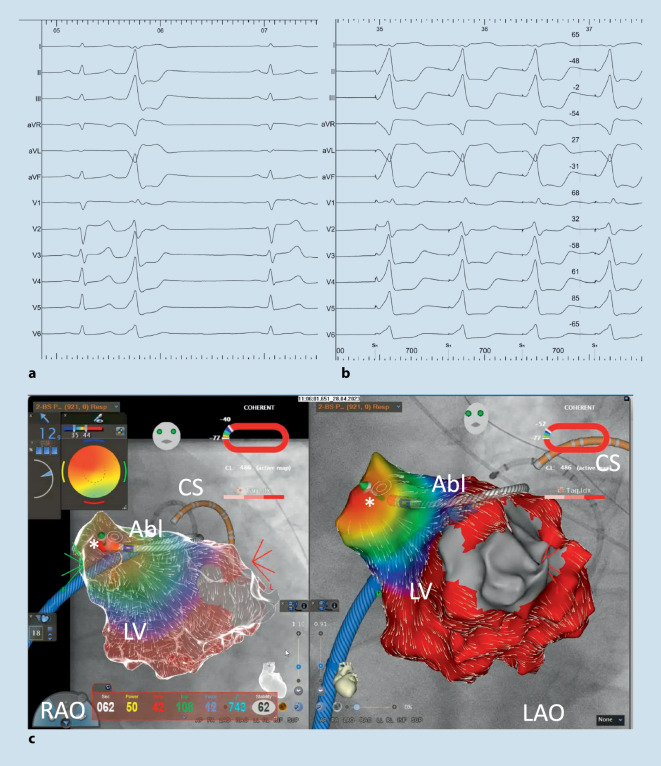

Mit neuen 3D-Mappingtechnologien wie High-density-Mapping mittels multipolarer Katheter sowie neuartigen Ablationskathetern mit Anpresskraftmessung und temperaturgesteuerter Ablation sowie steuerbaren Schleusen hat sich die Katheterablation von ventrikulären Arrhythmien unabhängig vom Vorliegen einer strukturellen Herzerkrankung deutlich verbessert (Abb. 4; [26, 27]). So liegt die Erfolgsrate der Katheterablation idiopathischer VT aus dem RVOT beispielsweise bei > 90 % und ist bei anderen Lokalisationen wie LVOT und Papillarmuskeln etwas geringer. Die Komplikationsraten sind mit < 1 % erfreulicherweise sehr niedrig [28]. Die aktuelle Entwicklung der sog. Pulse-field-Ablation (irreversible Elektroporation) mit ihrer extrem kurzen Applikationszeit und Kardiomyozytenselektivität wird hier sicherlich zukünftig noch weitere Verbesserungen hinsichtlich der Sicherheit und Effektivität liefern [29]. Erste Fallberichte bzgl. der Ablation ventrikulärer Arrhythmien aus dem RVOT sind vielversprechend und zeigen, dass die Erfolgsgeschichte der katheterbasierten Therapie von ventrikulären Arrhythmien (noch lange) nicht zu Ende ist [30].

## References

[1] Cronin EM, Bogun FM, Maury P, Peichl P, Chen M, Namboodiri N, Aguinaga L, Leite LR, Al-Khatib SM, Anter E, Berruezo A, Callans DJ, Chung MK, Cuculich P, Avila AD, Deal BJ, Deneke T, Dickfeld T-M, Hadid C, Haqqani HM, Kay GN, Latchamsetty R, Miller JM, Nogami A, Patel AR, Pathak RK, Morales LCS, Santangeli P, Sapp JL, Sarkozy A, Soejima K, Stevenson WG, Tedrow UB, Tzou WS, Varma N, Zeppenfeld K, Asirvatham SJ, Sternick EB, Chyou J, Ernst S, Fenelon G, Gerstenfeld EP, Hindricks G, Inoue K, Kim JJ, Krishnan K, Kuck K-H, Avalos MO, Paul T, Scanavacca MI, Tung R, Voss J, Yamada T, Yamane T, Group ESD (2019). 2019 HRS/EHRA/APHRS/LAHRS expert consensus statement on catheter ablation of ventricular arrhythmias. Europace.

[2] Breithardt G, Borggrefe M, Wichter T (1990). Catheter ablation of idiopathic right ventricular tachycardia. Circulation.

[3] Hasdemir C, Ulucan C, Yavuzgil O, Yuksel A, Kartal Y, Simsek E, Musayev O, Kayikcioglu M, Payzin S, Kultursay H, Aydin M, Can LH (2011). Tachycardia-induced cardiomyopathy in patients with idiopathic ventricular arrhythmias: the incidence, clinical and electrophysiologic characteristics, and the predictors. J Cardiovasc Electrophysiol.

[4] Massing MW, Simpson RJ, Rautaharju PM, Schreiner PJ, Crow R, Heiss G (2005). Usefulness of ventricular premature complexes to predict coronary heart disease events and mortality (from the Atherosclerosis Risk In Communities cohort). Am J Cardiol.

[5] Martini B, Nava A, Thiene G, Buja GF, Canciani B, Scognamiglio R, Daliento L, Volta SD (1989). Ventricular fibrillation without apparent heart disease: Description of six cases. Am Heart J.

[6] Brugada P, Brugada J (1992). Right bundle branch block, persistent ST segment elevation and sudden cardiac death: A distinct clinical and electrocardiographic syndrome A multicenter report. J Am Coll Cardiol.

[7] Antzelevitch C, Shimizu W (2002). Cellular mechanisms underlying the long QT syndrome. Curr Opin Cardiol.

[8] Zeppenfeld K, Tfelt-Hansen J, de Riva M, Winkel BG, Behr ER, Blom NA, Charron P, Corrado D, Dagres N, de Chillou C, Eckardt L, Friede T, Haugaa KH, Hocini M, Lambiase PD, Marijon E, Merino JL, Peichl P, Priori SG, Reichlin T, Schulz-Menger J, Sticherling C, Tzeis S, Verstrael A, Volterrani M, Cikes M, Kirchhof P, Abdelhamid M, Aboyans V, Arbelo E, Arribas F, Asteggiano R, Basso C, Bauer A, Bertaglia E, Biering-Sørensen T, Blomström-Lundqvist C, Borger MA, Čelutkienė J, Cosyns B, Falk V, Fauchier L, Gorenek B, Halvorsen S, Hatala R, Heidbuchel H, Kaab S, Konradi A, Koskinas KC, Kotecha D, Landmesser U, Lewis BS, Linhart A, Løchen ML, Lund LH, Metzner A, Mindham R, Nielsen JC, Norekvål TM, Patten M, Prescott E, Rakisheva A, Remme CA, Roca-Luque I, Sarkozy A, Scherr D, Sitges M, Touyz RM, Mieghem NV, Velagic V, Viskin S, Volders PGA, Kichou B, Martirosyan M, Scherr D, Aliyev F, Willems R, Naser N, Shalganov T, Milicic D, Christophides T, Kautzner J, Hansen J, Allam L, Kampus P, Junttila J, Leclercq C, Etsadashvili K, Steven D, Gatzoulis K, Gellér L, Arnar DO, Galvin J, Haim M, Pappone C, Elezi S, Kerimkulova A, Kalejs O, Rabah A, Puodziukynas A, Dimmer C, Sammut MA, David L, Boskovic A, Moustaghfir A, Maass AH, Poposka L, Mjolstad OC, Mitkowski P, Parreira L, Cozma D, Golukhova E, Bini R, Stojkovic S, Hlivak P, Pernat A, Castellano NP, Platonov PG, Duru F, Saadi ARA, Ouali S, Demircan S, Sychov O, Slade A (2022). 2022 ESC Guidelines for the management of patients with ventricular arrhythmias and the prevention of sudden cardiac death. Eur Heart J.

[9] Krittayaphong R, Bhuripanyo K, Punlee K, Kangkagate C, Chaithiraphan S (2002). Effect of atenolol on symptomatic ventricular arrhythmia without structural heart disease: A randomized placebo-controlled study. Am Heart J.

[10] Hoffmayer KS, Gerstenfeld EP (2013). Diagnosis and Management of Idiopathic Ventricular Tachycardia. Curr Probl Cardiol.

[11] Scheinman MA (2003). Reflections on the First Catheter Ablation of the Atrioventricular Junction. Pacing Clin Electrophysiol.

[12] Morady F, Sgheinman MM (1984). Transvenous Catheter Ablation of a Posteroseptal Accessory Pathway in a Patient with the Wolff-Parkinson-White Syndrome. N Engl J Med.

[13] Kuck KH, Kunze KP, Schlüter M, Geiger M, Jackman WM, Naccarelli GV (1988). Modification of a left-sided accessory atrioventricular pathway by radiofrequency current using a bipolar epicardial-endocardial electrode configuration. Eur Heart J.

[14] Kuck K, Jackman WM, Pitha J, Kunzk K, Carmen L, Schröder S, Nienaber CA (1988). Percutaneous Catheter Ablation at the Mitral Annulus in Canines Using a Bipolar Epicardial-Endocardial Electrode Configuration. Pacing Clin Electrophysiol.

[15] Kuck K-H, Schluter M, Geiger M, Siebels J, Duckeck W (1991). Radiofrequency current catheter ablation of accessory atrioventricular pathways. Lancet.

[16] Zhu DW-X, Maloney JD, Simmons TW, Nitta J, Fitzgerald DM, Trohman RG, Khoury DS, Saliba W, Belco KM, Rizo-Patron C, Pinski SL (1995). Radiofrequency catheter ablation for management of symptomatic ventricular ectopic activity. J Am Coll Cardiol.

[17] Klein LS, Shih HT, Hackett FK, Zipes DP, Miles WM (1992). Radiofrequency catheter ablation of ventricular tachycardia in patients without structural heart disease. Circulation.

[18] Wellens HJJ (1995). Radiofrequency catheter ablation of benign ventricular ectopic beats: A therapy in search of a disease?. J Am Coll Cardiol.

[19] Chun KRJ, Satomi K, Kuck K-H, Ouyang F, Antz M (2007). Left Ventricular Outflow Tract Tachycardia Including Ventricular Tachycardia from the Aortic Cusps and Epicardial Ventricular Tachycardia. Herz.

[20] Shimoike E, Ohnishi Y, Ueda N, Maruyama T, Kaji Y (1999). Radiofrequency Catheter Ablation of Left Ventricular Outflow Tract Tachycardia from the Coronary Cusp: A New Approach to the Tachycardia Focus. J Cardiovasc Electrophysiol.

[21] Ouyang F, Fotuhi P, Ho SY, Hebe J, Volkmer M, Goya M, Burns M, Antz M, Ernst S, Cappato R, Kuck K-H (2002). Repetitive monomorphic ventricular tachycardia originating from the aortic sinus cusp Electrocardiographic characterization for guiding catheter ablation. J Am Coll Cardiol.

[22] Ouyang F, Mathew S, Wu S, Kamioka M, Metzner A, Xue Y, Ju W, Yang B, Zhan X, Rillig A, Lin T, Rausch P, Deiß S, Lemes C, Tönnis T, Wissner E, Tilz RR, Kuck K-H, Chen M (2014). Ventricular Arrhythmias Arising From the Left Ventricular Outflow Tract Below the Aortic Sinus Cusps: Mapping and Catheter Ablation via Transseptal Approach and Electrocardiographic Characteristics. Circ Arrhythm Electrophysiol.

[23] Heeger C-H, Hayashi K, Kuck K-H, Ouyang F (2016). Catheter Ablation of Idiopathic Ventricular Arrhythmias Arising From the Cardiac Outflow Tracts—Recent Insights and Techniques for the Successful Treatment of Common and Challenging Cases. Circ J.

[24] Heeger C, Kuck K, Ouyang F (2017). Catheter ablation of pulmonary sinus cusp-derived ventricular arrhythmias by the reversed U-curve technique. J Cardiovasc Electrophysiol.

[25] Liao Z, Zhan X, Wu S, Xue Y, Fang X, Liao H, Deng H, Liang Y, Wei W, Liu Y, Ouyang F (2015). Idiopathic Ventricular Arrhythmias Originating From the Pulmonary Sinus Cusp Prevalence, Electrocardiographic/Electrophysiological Characteristics, and Catheter Ablation. J Am Coll Cardiol.

[26] Heeger C-H, Mamaev R, Eitel C, Kuck K-H, Tilz RR (2023). Treatment of frequent premature ventricular contractions via a single very high-power short-duration application. Europace.

[27] Heeger C-H, Popescu SS, Kirstein B, Hatahet S, Traub A, Phan H-L, Feher M, D’Ambrosio G, Keelani A, Schlüter M, Vogler J, Eitel C, Kuck K-H, Tilz RR (2022). Very-high-power short-duration ablation for treatment of premature ventricular contractions—The Fast-And-Furious PVC study. Int J Cardiol Heart Vasc.

[28] Tokuda M, Kojodjojo P, Epstein LM, Koplan BA, Michaud GF, Tedrow UB, Stevenson WG, John RM (2011). Outcomes of cardiac perforation complicating catheter ablation of ventricular arrhythmias. Circ Arrhythm Electrophysiol.

[29] Turagam MK, Neuzil P, Schmidt B, Reichlin T, Neven K, Metzner A, Hansen J, Blaauw Y, Maury P, Arentz T, Sommer P, Anic A, Anselme F, Boveda S, Deneke T, Willems S, van der Voort P, Tilz R, Funasako M, Scherr D, Wakili R, Steven D, Kautzner J, Vijgen J, Jais P, Petru J, Chun J, Roten L, Füting A, Lemoine MD, Ruwald M, Mulder BA, Rollin A, Lehrmann H, Fink T, Jurisic Z, Chaumont C, Adeliño R, Nentwich K, Gunawardene M, Ouss A, Heeger C-H, Manninger M, Bohnen J-E, Sultan A, Peichl P, Koopman P, Derval N, Kueffer T, Rahe G, Reddy VY (2023). Safety and Effectiveness of Pulsed Field Ablation to Treat Atrial Fibrillation: One-Year Outcomes From the MANIFEST-PF Registry. Circulation.

[30] Schmidt B, Chen S, Tohoku S, Bordignon S, Bologna F, Chun KRJ (2021). Single shot electroporation of premature ventricular contractions from the right ventricular outflow tract. Europace.

